# Crystal structure of 6,7-de­hydro­royleanone isolated from *Taxodium distichum* (L.) Rich.

**DOI:** 10.1107/S2056989017017935

**Published:** 2018-01-01

**Authors:** Li Chen, Xinhua Ma, ShiHao Deng, XinZhou Yang, Ping Song

**Affiliations:** a School of Pharmaceutical Sciences, South-Central University for Nationalities, Wuhan 430074, People’s Republic of China; b College of Chemistry and Life Science, Qinghai University for Nationalities, Xining, 810007, People’s Republic of China

**Keywords:** crystal structure, 6,7-de­hydro­royleanone, *Taxodium distichum* (*L*.) Rich

## Abstract

The title compound, 6,7-de­hydro­royleanone, C_20_H_26_O_3_ [systematic name: (4b*S*)-3-hy­droxy-2-isopropyl-4b,8,8-trimethyl-4b,5,6,7,8,8a-hexa­hydro­phenanthrene-1,4-dione] was isolated from *Taxodium distichum* (L.) Rich. The compound crystallizes in the space group *P*2_1_. The crystal structure features two O—H⋯O hydrogen bonds, forming chains along the [010] direction.

## Abstract 2

The crystal structure features two O—H⋯O hydrogen bonds, forming chains along the [010] direction.

## Chemical context   

*Taxodium distichum* (L.) Rich. is a tree native to North America that can grow to 25 m in height (Ogunwande *et al.*, 2007 ▸). Its leaves and seeds are used for the treatment of malaria and liver disease (Kupchan *et al.*, 1968 ▸). Previous studies revealed that it contains multiple compounds such as diterpenes (Kusumoto *et al.*, 2010 ▸), flavonoids (Zaghloul *et al.*, 2008 ▸), proanthocyanidins (Stafford & Lester, 1986 ▸), lignins (Logan & Thomas, 1985 ▸), sterols and fatty acids (Geiger & de Groot-Pfleiderer, 1979 ▸). A detailed phytochemical investigation of a petroleum ether extract of the seeds of *Taxodium distichum* (L.) Rich. led to the isolation of the title compound 6,7-de­hydro­royleanone. Herein we present the crystal structure of 6,7-de­hydro­royleanone, which was undertaken in order to establish unambiguously the stereochemical features of this natural compound.
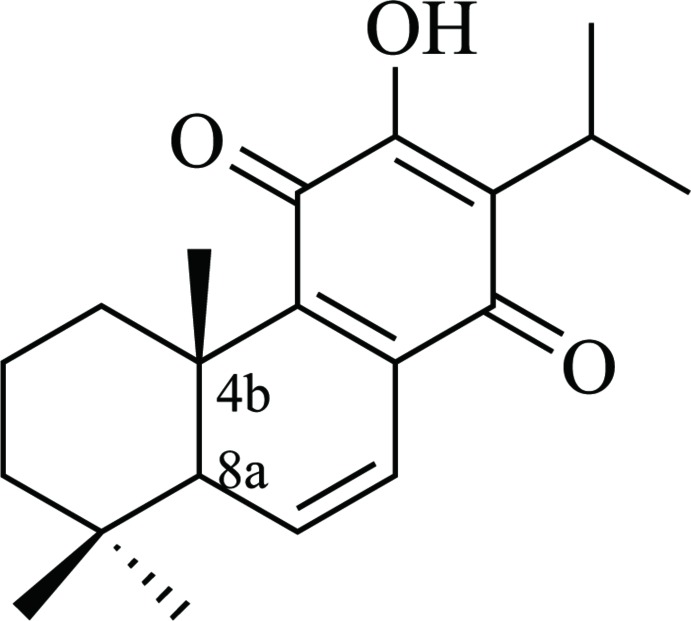


## Structural commentary   

**Figure 1:**
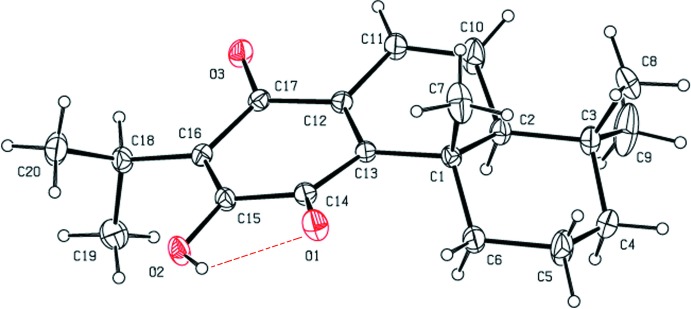
The mol­ecular structure of the title compound, with the atom labelling and 50% probability displacement ellipsoids. The intra­molecular O—H⋯O hydrogen bond (see Table 1 ▸) is shown as a red dashed line.

The mol­ecular structure of the title compound is shown in Fig. 1 ▸. The title compound belongs to the class of abietane-type diterpenes and the structure contains two ketone groups at C14 and C17 and three double bonds located between atoms C10 and C11, C12 and C13, C15 and C16. The torsion angles C17—C12—C13—C1 [176.8 (2)°], C11—C12—C13—C14 [168.7 (3)°], C6—C1—C2—C10 [171.6 (3)°] and C13—C1—C2—C3 [−173.4 (3)°] describe the geometry at the junctions of the three rings. An intra­molecular O2—H2*A*⋯O1 hydrogen bond (Table 1 ▸) stabilizes the mol­ecular conformation.

## Supra­molecular features   

**Figure 2:**
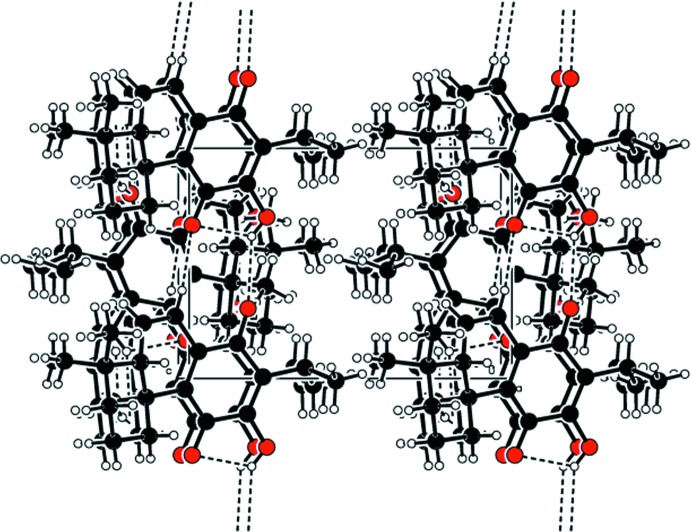
Part of the crystal structure of the title compound, with hydrogen bonds (see Table 1 ▸) shown as dashed lines.

In the crystal, O2—H*2A*⋯O3^i^ and C11—H11⋯O1^i^ hydrogen bonds link the mol­ecules, forming chains along [010] (Table 1 ▸ and Fig. 2 ▸).

## Database survey   

A search of the Cambridge Structural Database (CSD, Version 5.27, last update Feb 2017; Groom *et al.*, 2016 ▸) yielded the compound royleanone (HACGUN01; Fun *et al.*, 2011 ▸), which has a similar structure to the title compound but without the double bond between C10 and C11.

## Synthesis and crystallization   

The title compound was isolated from the seeds of *Taxodium distichum* (L.) Rich. collected in Xining, China, in April 2015 (SC0185). The air-dried seeds of *Taxodium distichum* (1.1 kg) were extracted with 95% EtOH and then partitioned successively with petroleum ether (PE), ethyl acetate (EtOAc) and *n*-butyl alcohol (*n*-BuOH) to give a PE extract (30 g), an EtOAc extract (50 g) and an *n*-BuOH extract (68 g). The PE extract (30 g) was subjected to normal-phase silica-gel column chromatography (300–400 mesh) with a gradient solvent system of petroleum ether–ethyl acetate (1:0-0:1, *v*/*v*, containing 0.1% formic acid) to give ten major fractions, denoted F1–F10. F7 (2.8 g) was sequentially subjected to Sephadex-LH20 gel column chromatography (CH_2_Cl_2_–MeOH, 3:1, *v*/*v*, containing 0.1% formic acid) to give four major fractions F7.1–F7.4. F7.3 was purified by semi-preparative HPLC (CNCH_3_/H_2_O, 20:80→100:0, 40 min, containing 0.1% formic acid in both phases) to give an orange solid, which was recrystallized from a solvent mix of CH_2_Cl_2_–MeOH (5:1) affording orange block-like crystals suitable for X-ray diffraction analysis. For the ^1^H and ^13^C NMR data of 6,7-de­hydro­royleanone, see Chang *et al.* (2001 ▸).

## Refinement   

Crystal data, data collection and structure refinement details are summarized in Table 2 ▸. C-bound H atoms were positioned with idealized geometry and refined isotropically using a riding model with C—H = 0.94–0.99 Å and *U*_iso_(H) = 1.5*U*_eq_(C) for methyl H atoms and 1.2*U*_eq_(C) for all others. The OH hydrogen atom was refined freely with *U*_iso_(H) = 1.5*U*_eq_(O).

## Supplementary Material

Crystal structure: contains datablock(s) I. DOI: 10.1107/S2056989017017935/qm2118sup1.cif

Structure factors: contains datablock(s) I. DOI: 10.1107/S2056989017017935/qm2118Isup2.hkl

Supporting information file. DOI: 10.1107/S2056989017017935/qm2118Isup3.cdx

Supporting information file. DOI: 10.1107/S2056989017017935/qm2118Isup4.cml

CCDC reference: 1551127

Additional supporting information:  crystallographic information; 3D view; checkCIF report

## Figures and Tables

**Table 1:** Hydrogen-bond geometry (Å, °)

*D*—H⋯*A*	*D*—H	H⋯*A*	*D*⋯*A*	*D*—H⋯*A*
O2—H2*A*⋯O1	0.82 (6)	2.03 (5)	2.607 (3)	128 (5)
O2—H2*A*⋯O3^i^	0.82 (6)	2.53 (6)	3.160 (3)	135 (5)
C11—H11⋯O1^ii^	0.93	2.37	3.290 (3)	173 (5)

Symmetry codes: (i) 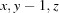; (ii) 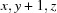.

**Table 2:** Experimental details

Crystal data	
Chemical formula	C_20_H_26_O_3_
*M*_r_	314.41
Crystal system, space group	Monoclinic, *P*2_1_
Temperature (K)	296
*a*, *b*, *c* (Å)	10.4348 (17), 7.6726 (13), 10.8210 (18)
β (°)	97.773 (3)
*V* (Å^3^)	858.4 (2)
*Z*	2
Radiation type	Mo *K*α
μ (mm^−1^)	0.08
Crystal size (mm)	0.3 × 0.2 × 0.2

Data collection	
Diffractometer	Bruker P4
No. of measured, independent and observed [*I* > 2σ(*I*)] reflections	6718, 3416, 2980
*R*_int_	0.022
(sin θ/λ)_max_ (Å^−1^)	0.625

Refinement	
*R*[*F* ^2^ > 2σ(*F* ^2^)], *wR*(*F* ^2^), *S*	0.047, 0.143, 1.08
No. of reflections	3416
No. of parameters	216
No. of restraints	1
H-atom treatment	H atoms treated by a mixture of independent and constrained refinement
Δρ_max_, Δρ_min_ (e Å^−3^)	0.27, −0.18

Computer programs: *APEX2* and *SAINT* (Bruker, 2007 ▸), *SHELXT* (Sheldrick, 2015*a* ▸), *SHELXL2016* (Sheldrick, 2015*b* ▸) and *OLEX2* (Dolomanov *et al.*, 2009 ▸).

## supplementary crystallographic information

### Crystal data

**Table:**

C_20_H_26_O_3_	*F*(000) = 340
*M**_r_* = 314.41	*D*_x_ = 1.216 Mg m^−^^3^
Monoclinic, *P*2_1_	Mo *K*α radiation, λ = 0.71073 Å
*a* = 10.4348 (17) Å	Cell parameters from 2696 reflections
*b* = 7.6726 (13) Å	θ = 2.6–27.3°
*c* = 10.8210 (18) Å	µ = 0.08 mm^−^^1^
β = 97.773 (3)°	*T* = 296 K
*V* = 858.4 (2) Å^3^	Block, orange
*Z* = 2	0.3 × 0.2 × 0.2 mm

### Data collection

**Table:**

Bruker P4 diffractometer	θ_max_ = 26.4°, θ_min_ = 1.9°
φ and ω scans	*h* = −13→12
6718 measured reflections	*k* = −9→9
3416 independent reflections	*l* = −13→13
2980 reflections with *I* > 2σ(*I*)	1 standard reflections every 300 reflections
*R*_int_ = 0.022	intensity decay: 1%

### Refinement

**Table:**

Refinement on *F*^2^	1 restraint
Least-squares matrix: full	Hydrogen site location: mixed
*R*[*F*^2^ > 2σ(*F*^2^)] = 0.047	H atoms treated by a mixture of independent and constrained refinement
*wR*(*F*^2^) = 0.143	*w* = 1/[σ^2^(*F*_o_^2^) + (0.0865*P*)^2^ + 0.0799*P*] where *P* = (*F*_o_^2^ + 2*F*_c_^2^)/3
*S* = 1.08	(Δ/σ)_max_ = 0.003
3416 reflections	Δρ_max_ = 0.27 e Å^−^^3^
216 parameters	Δρ_min_ = −0.18 e Å^−^^3^

### Special details

**Table:**

Geometry. All esds (except the esd in the dihedral angle between two l.s. planes) are estimated using the full covariance matrix. The cell esds are taken into account individually in the estimation of esds in distances, angles and torsion angles; correlations between esds in cell parameters are only used when they are defined by crystal symmetry. An approximate (isotropic) treatment of cell esds is used for estimating esds involving l.s. planes.

### Fractional atomic coordinates and isotropic or equivalent isotropic displacement parameters (Å^2^)

**Table:**

	*x*	*y*	*z*	*U*_iso_*/*U*_eq_	
C1	0.3123 (3)	0.9175 (4)	0.1442 (2)	0.0325 (6)	
C2	0.4090 (3)	1.0711 (4)	0.1650 (3)	0.0400 (7)	
H2	0.454767	1.067917	0.091644	0.048*	
C3	0.5186 (3)	1.0545 (5)	0.2774 (3)	0.0471 (8)	
C4	0.5860 (4)	0.8794 (7)	0.2667 (4)	0.0712 (13)	
H4A	0.633484	0.882840	0.195663	0.085*	
H4B	0.647861	0.861196	0.340907	0.085*	
C5	0.4925 (6)	0.7270 (6)	0.2515 (5)	0.098 (2)	
H5A	0.450167	0.716784	0.325687	0.117*	
H5B	0.540153	0.620204	0.242845	0.117*	
C6	0.3896 (4)	0.7501 (5)	0.1370 (4)	0.0696 (13)	
H6A	0.431584	0.752332	0.062273	0.084*	
H6B	0.331142	0.651222	0.130827	0.084*	
C7	0.2214 (4)	0.9005 (8)	0.2443 (3)	0.0746 (14)	
H7A	0.148629	0.829236	0.213057	0.112*	
H7B	0.191762	1.014064	0.264675	0.112*	
H7C	0.267276	0.847719	0.317782	0.112*	
C8	0.4756 (5)	1.0761 (7)	0.4025 (4)	0.0797 (14)	
H8A	0.427134	0.975447	0.421190	0.120*	
H8B	0.422203	1.178020	0.402234	0.120*	
H8C	0.549959	1.088847	0.464619	0.120*	
C9	0.6187 (5)	1.1986 (9)	0.2651 (6)	0.106 (2)	
H9A	0.581634	1.310139	0.279235	0.160*	
H9B	0.643405	1.195624	0.182830	0.160*	
H9C	0.693627	1.179893	0.325620	0.160*	
C10	0.3398 (5)	1.2400 (6)	0.1542 (5)	0.0861 (17)	
H10	0.356742	1.324207	0.216089	0.103*	
C11	0.2507 (3)	1.2703 (4)	0.0517 (3)	0.0480 (8)	
H11	0.222528	1.382398	0.029548	0.058*	
C12	0.2011 (3)	1.1170 (4)	−0.0221 (3)	0.0346 (6)	
C13	0.2247 (3)	0.9533 (3)	0.0209 (2)	0.0312 (6)	
C14	0.1516 (3)	0.8125 (4)	−0.0477 (3)	0.0370 (6)	
C15	0.0868 (3)	0.8459 (4)	−0.1776 (3)	0.0376 (7)	
C16	0.0709 (3)	1.0066 (4)	−0.2261 (3)	0.0359 (6)	
C17	0.1215 (3)	1.1519 (4)	−0.1454 (3)	0.0346 (6)	
C18	0.0096 (3)	1.0468 (5)	−0.3588 (3)	0.0451 (7)	
H18	0.001388	1.173803	−0.365603	0.054*	
C19	0.0958 (4)	0.9899 (7)	−0.4517 (3)	0.0680 (11)	
H19A	0.177527	1.048720	−0.434859	0.102*	
H19B	0.055809	1.018277	−0.534386	0.102*	
H19C	0.109290	0.866270	−0.445188	0.102*	
C20	−0.1270 (4)	0.9706 (7)	−0.3885 (4)	0.0711 (12)	
H20A	−0.121950	0.845769	−0.390726	0.107*	
H20B	−0.166517	1.012984	−0.468077	0.107*	
H20C	−0.177917	1.005487	−0.325198	0.107*	
O1	0.1376 (3)	0.6667 (3)	−0.0048 (2)	0.0561 (7)	
O2	0.0435 (3)	0.7012 (3)	−0.2393 (2)	0.0521 (6)	
H2A	0.058 (5)	0.628 (8)	−0.184 (5)	0.078*	
O3	0.1021 (2)	1.3039 (3)	−0.1790 (2)	0.0497 (6)	

### Atomic displacement parameters (Å^2^)

**Table:**

	*U*^11^	*U*^22^	*U*^33^	*U*^12^	*U*^13^	*U*^23^
C1	0.0388 (14)	0.0315 (15)	0.0263 (12)	−0.0009 (12)	0.0013 (10)	0.0016 (10)
C2	0.0381 (14)	0.0392 (17)	0.0405 (14)	−0.0066 (13)	−0.0029 (11)	0.0027 (13)
C3	0.0432 (16)	0.0488 (19)	0.0448 (16)	−0.0042 (15)	−0.0108 (13)	0.0022 (15)
C4	0.060 (2)	0.086 (3)	0.060 (2)	0.027 (2)	−0.0208 (18)	−0.012 (2)
C5	0.119 (4)	0.047 (3)	0.105 (4)	0.026 (3)	−0.070 (3)	−0.010 (3)
C6	0.085 (3)	0.043 (2)	0.069 (2)	0.022 (2)	−0.035 (2)	−0.0170 (18)
C7	0.056 (2)	0.126 (4)	0.0416 (19)	−0.030 (2)	0.0079 (16)	0.008 (2)
C8	0.082 (3)	0.101 (4)	0.050 (2)	0.002 (3)	−0.0137 (19)	−0.031 (2)
C9	0.074 (3)	0.113 (5)	0.116 (4)	−0.049 (3)	−0.047 (3)	0.047 (4)
C10	0.098 (3)	0.034 (2)	0.107 (4)	−0.002 (2)	−0.058 (3)	−0.010 (2)
C11	0.0561 (18)	0.0279 (16)	0.0545 (19)	−0.0007 (13)	−0.0124 (15)	−0.0005 (14)
C12	0.0351 (13)	0.0292 (14)	0.0383 (14)	−0.0001 (11)	0.0009 (11)	0.0001 (12)
C13	0.0334 (13)	0.0278 (15)	0.0318 (13)	−0.0006 (11)	0.0022 (10)	0.0009 (11)
C14	0.0433 (15)	0.0285 (16)	0.0377 (15)	−0.0001 (12)	0.0003 (12)	0.0015 (12)
C15	0.0410 (14)	0.0318 (16)	0.0375 (15)	−0.0029 (12)	−0.0038 (12)	−0.0037 (12)
C16	0.0352 (14)	0.0364 (16)	0.0348 (14)	0.0040 (11)	0.0000 (11)	0.0021 (12)
C17	0.0321 (13)	0.0309 (15)	0.0403 (15)	0.0017 (12)	0.0026 (11)	0.0016 (12)
C18	0.0541 (18)	0.0399 (18)	0.0372 (15)	0.0030 (15)	−0.0087 (13)	0.0022 (13)
C19	0.084 (3)	0.083 (3)	0.0374 (16)	0.013 (2)	0.0072 (17)	0.0045 (19)
C20	0.058 (2)	0.072 (3)	0.074 (3)	0.002 (2)	−0.025 (2)	−0.002 (2)
O1	0.0790 (16)	0.0311 (12)	0.0532 (14)	−0.0106 (11)	−0.0091 (12)	0.0062 (10)
O2	0.0703 (15)	0.0339 (13)	0.0454 (12)	−0.0065 (11)	−0.0170 (11)	−0.0030 (10)
O3	0.0576 (13)	0.0312 (12)	0.0554 (14)	0.0020 (10)	−0.0102 (11)	0.0077 (10)

### Geometric parameters (Å, º)

**Table:**

C1—C2	1.548 (4)	C9—H9C	0.9600
C1—C6	1.525 (5)	C10—H10	0.9300
C1—C7	1.538 (4)	C10—C11	1.367 (5)
C1—C13	1.537 (4)	C11—H11	0.9300
C2—H2	0.9800	C11—C12	1.475 (4)
C2—C3	1.558 (4)	C12—C13	1.350 (4)
C2—C10	1.481 (6)	C12—C17	1.497 (4)
C3—C4	1.528 (6)	C13—C14	1.466 (4)
C3—C8	1.492 (5)	C14—C15	1.497 (4)
C3—C9	1.539 (6)	C14—O1	1.227 (4)
C4—H4A	0.9700	C15—C16	1.341 (4)
C4—H4B	0.9700	C15—O2	1.342 (4)
C4—C5	1.517 (8)	C16—C17	1.470 (4)
C5—H5A	0.9700	C16—C18	1.522 (4)
C5—H5B	0.9700	C17—O3	1.230 (4)
C5—C6	1.536 (5)	C18—H18	0.9800
C6—H6A	0.9700	C18—C19	1.501 (5)
C6—H6B	0.9700	C18—C20	1.534 (5)
C7—H7A	0.9600	C19—H19A	0.9600
C7—H7B	0.9600	C19—H19B	0.9600
C7—H7C	0.9600	C19—H19C	0.9600
C8—H8A	0.9600	C20—H20A	0.9600
C8—H8B	0.9600	C20—H20B	0.9600
C8—H8C	0.9600	C20—H20C	0.9600
C9—H9A	0.9600	O2—H2A	0.82 (6)
C9—H9B	0.9600		
			
C6—C1—C2	108.0 (3)	C3—C9—H9B	109.5
C6—C1—C7	110.2 (4)	C3—C9—H9C	109.5
C6—C1—C13	111.3 (2)	H9A—C9—H9B	109.5
C7—C1—C2	114.5 (3)	H9A—C9—H9C	109.5
C13—C1—C2	106.8 (2)	H9B—C9—H9C	109.5
C13—C1—C7	106.0 (2)	C2—C10—H10	120.6
C1—C2—H2	104.1	C11—C10—C2	118.9 (4)
C1—C2—C3	116.5 (3)	C11—C10—H10	120.6
C3—C2—H2	104.1	C10—C11—H11	121.5
C10—C2—C1	110.7 (3)	C10—C11—C12	117.0 (3)
C10—C2—H2	104.1	C12—C11—H11	121.5
C10—C2—C3	115.5 (3)	C11—C12—C17	116.8 (2)
C4—C3—C2	108.0 (3)	C13—C12—C11	121.4 (3)
C4—C3—C9	107.5 (4)	C13—C12—C17	121.8 (2)
C8—C3—C2	114.9 (3)	C12—C13—C1	121.7 (2)
C8—C3—C4	111.3 (4)	C12—C13—C14	116.9 (2)
C8—C3—C9	106.8 (4)	C14—C13—C1	121.0 (2)
C9—C3—C2	108.0 (3)	C13—C14—C15	119.2 (2)
C3—C4—H4A	109.0	O1—C14—C13	124.1 (3)
C3—C4—H4B	109.0	O1—C14—C15	116.8 (3)
H4A—C4—H4B	107.8	C16—C15—C14	122.8 (3)
C5—C4—C3	113.0 (3)	C16—C15—O2	123.4 (3)
C5—C4—H4A	109.0	O2—C15—C14	113.8 (3)
C5—C4—H4B	109.0	C15—C16—C17	116.7 (2)
C4—C5—H5A	109.3	C15—C16—C18	124.7 (3)
C4—C5—H5B	109.3	C17—C16—C18	118.6 (3)
C4—C5—C6	111.6 (4)	C16—C17—C12	120.4 (2)
H5A—C5—H5B	108.0	O3—C17—C12	118.8 (3)
C6—C5—H5A	109.3	O3—C17—C16	120.8 (3)
C6—C5—H5B	109.3	C16—C18—H18	107.1
C1—C6—C5	111.9 (3)	C16—C18—C20	112.3 (3)
C1—C6—H6A	109.2	C19—C18—C16	111.0 (3)
C1—C6—H6B	109.2	C19—C18—H18	107.1
C5—C6—H6A	109.2	C19—C18—C20	111.9 (3)
C5—C6—H6B	109.2	C20—C18—H18	107.1
H6A—C6—H6B	107.9	C18—C19—H19A	109.5
C1—C7—H7A	109.5	C18—C19—H19B	109.5
C1—C7—H7B	109.5	C18—C19—H19C	109.5
C1—C7—H7C	109.5	H19A—C19—H19B	109.5
H7A—C7—H7B	109.5	H19A—C19—H19C	109.5
H7A—C7—H7C	109.5	H19B—C19—H19C	109.5
H7B—C7—H7C	109.5	C18—C20—H20A	109.5
C3—C8—H8A	109.5	C18—C20—H20B	109.5
C3—C8—H8B	109.5	C18—C20—H20C	109.5
C3—C8—H8C	109.5	H20A—C20—H20B	109.5
H8A—C8—H8B	109.5	H20A—C20—H20C	109.5
H8A—C8—H8C	109.5	H20B—C20—H20C	109.5
H8B—C8—H8C	109.5	C15—O2—H2A	101 (4)
C3—C9—H9A	109.5		
			
C1—C2—C3—C4	52.3 (4)	C11—C12—C13—C14	168.7 (3)
C1—C2—C3—C8	−72.7 (4)	C17—C12—C13—C1	176.8 (2)
C1—C2—C3—C9	168.2 (4)	C17—C12—C13—C14	−10.0 (4)
C1—C2—C10—C11	−50.7 (6)	C11—C12—C17—C16	178.5 (3)
C1—C13—C14—C15	−169.1 (2)	C11—C12—C17—O3	0.3 (4)
C1—C13—C14—O1	12.1 (4)	C12—C13—C14—C15	17.7 (4)
C2—C1—C6—C5	54.3 (5)	C12—C13—C14—O1	−161.1 (3)
C2—C1—C13—C12	−27.2 (3)	C13—C1—C2—C3	−173.4 (3)
C2—C1—C13—C14	159.9 (2)	C13—C1—C2—C10	51.8 (4)
C2—C3—C4—C5	−52.3 (4)	C13—C1—C6—C5	171.2 (4)
C2—C10—C11—C12	17.7 (7)	C13—C12—C17—C16	−2.5 (4)
C3—C2—C10—C11	174.0 (4)	C13—C12—C17—O3	179.2 (3)
C3—C4—C5—C6	57.4 (6)	C13—C14—C15—C16	−13.2 (4)
C4—C5—C6—C1	−58.2 (6)	C13—C14—C15—O2	168.3 (3)
C6—C1—C2—C3	−53.7 (4)	C14—C15—C16—C17	0.4 (4)
C6—C1—C2—C10	171.6 (3)	C14—C15—C16—C18	178.0 (3)
C6—C1—C13—C12	−144.9 (3)	C15—C16—C17—C12	7.6 (4)
C6—C1—C13—C14	42.2 (4)	C15—C16—C17—O3	−174.3 (3)
C7—C1—C2—C3	69.5 (4)	C15—C16—C18—C19	−70.0 (4)
C7—C1—C2—C10	−65.2 (4)	C15—C16—C18—C20	56.2 (4)
C7—C1—C6—C5	−71.4 (5)	C17—C12—C13—C1	176.7 (2)
C7—C1—C13—C12	95.2 (4)	C17—C12—C13—C14	−10.1 (4)
C7—C1—C13—C14	−77.6 (4)	C17—C16—C18—C19	107.6 (3)
C8—C3—C4—C5	74.7 (4)	C17—C16—C18—C20	−126.3 (3)
C9—C3—C4—C5	−168.6 (4)	C18—C16—C17—C12	−170.2 (2)
C10—C2—C3—C4	−175.2 (4)	C18—C16—C17—O3	8.0 (4)
C10—C2—C3—C8	59.9 (5)	O1—C14—C15—C16	165.6 (3)
C10—C2—C3—C9	−59.2 (5)	O1—C14—C15—O2	−12.8 (4)
C10—C11—C12—C13	11.3 (5)	O2—C15—C16—C17	178.7 (3)
C10—C11—C12—C17	−169.8 (4)	O2—C15—C16—C18	−3.8 (5)
C11—C12—C13—C1	−4.4 (4)		

### Hydrogen-bond geometry (Å, º)

**Table:**

*D*—H···*A*	*D*—H	H···*A*	*D*···*A*	*D*—H···*A*
O2—H2*A*···O1	0.82 (6)	2.03 (5)	2.607 (3)	128 (5)
O2—H2*A*···O3^i^	0.82 (6)	2.53 (6)	3.160 (3)	135 (5)
C11—H11···O1^ii^	0.93	2.37	3.290 (3)	173 (5)

Symmetry codes: (i) *x*, *y*−1, *z*; (ii) *x*, *y*+1, *z*.
